# Ferric Citrate Uptake Is a Virulence Factor in Uropathogenic Escherichia coli

**DOI:** 10.1128/mbio.01035-22

**Published:** 2022-05-12

**Authors:** Arwen E. Frick-Cheng, Anna Sintsova, Sara N. Smith, Ali Pirani, Evan S. Snitkin, Harry L. T. Mobley

**Affiliations:** a Department of Microbiology and Immunology, University of Michigan, Ann Arbor, Michigan, USA; University of Chicago

**Keywords:** urinary tract infection, *Escherichia coli*, iron acquisition, virulence factors, pathogenesis, siderophores, iron transport

## Abstract

More than half of women will experience a urinary tract infection (UTI), with uropathogenic Escherichia coli (UPEC) causing ~80% of uncomplicated cases. Iron acquisition systems are essential for uropathogenesis, and UPEC strains encode highly diverse iron acquisition systems, underlining their importance. However, a recent UPEC clinical isolate, HM7, lacks this diversity and instead encodes the synthesis pathway for a sole siderophore, enterobactin. To determine if HM7 possesses unidentified iron acquisition systems, we performed RNA sequencing under iron-limiting conditions and demonstrated that the ferric citrate uptake system (*fecABCDE* and *fecIR*) was highly upregulated. Importantly, there are high levels of citrate within urine, some of which is bound to iron, and the *fec* system is enriched in UPEC isolates compared to fecal strains. Therefore, we hypothesized that HM7 and other similar strains use the *fec* system to acquire iron in the host. Deletion of both enterobactin biosynthesis and ferric citrate uptake (Δ*fecA*/Δ*entB*) abrogates use of ferric citrate as an iron source, and *fecA* provides an advantage in human urine in the absence of enterobactin. However, in a UTI mouse model, *fecA* is a fitness factor independent of enterobactin production, likely due to the action of host lipocalin-2 chelating ferrienterobactin. These findings indicate that ferric citrate uptake is used as an iron source when siderophore efficacy is limited, such as in the host during UTI. Defining these novel compensatory mechanisms and understanding the nutritional hierarchy of preferred iron sources within the urinary tract are important in the search for new approaches to combat UTI.

## INTRODUCTION

More than half of women will experience a urinary tract infection (UTI) during their lifetime (1, 2), with uropathogenic Escherichia coli (UPEC) causing 80% of uncomplicated cases (3, 4). These infections are responsible for an annual five billion dollars of health care costs in the United States (5, 6). To survive within the host, UPEC encodes a wide array of virulence factors that include toxins, adhesins, and iron acquisition systems (6–9). Iron is an essential cofactor for many biological processes, including DNA replication, DNA repair, and central metabolism (10, 11). UPEC encodes several transporters to import free iron, such as the Sit, Feo, and Efe systems (12). However, mammalian hosts employ “nutritional immunity,” wherein iron is sequestered within proteins or molecules such as transferrin, lactoferrin, ferritin, and hemoglobin and is not readily accessible to bacteria via free iron transporters (13, 14). To survive in the host, UPEC has evolved mechanisms to acquire iron from sequestered sources, which fall into two broad categories: heme receptors and siderophores. Heme receptors import heme, allowing the bacteria to utilize the bound iron, while siderophores are small molecules with extraordinarily high affinities for iron (dissociation constants [*K_d_*] ranging from 10^23^ to 10^52^ M^−1^) (15, 16), which allow them to strip iron from sequestered sources.

UPEC can encode up to five systems that can acquire iron from sequestered sources: heme receptors (ChuA and Hma) and four siderophores (enterobactin, salmochelin, aerobactin, and yersiniabactin) (17–19). UPEC strains often employ a subset of these systems. For example, prototypical UPEC strain CFT073 encodes heme receptors and produces enterobactin, salmochelin, and aerobactin. This high level of diversity is essential for UPEC survival within the host due in part to specific host defenses. For instance, the innate immune protein lipocalin-2 (Lcn2) binds ferric and aferric enterobactin, preventing the bacterium from utilizing this siderophore (20). Therefore, UPEC cannot rely upon a single method of iron acquisition.

While heme receptors and siderophores are virulence-associated iron acquisition systems utilized by UPEC and other pathogenic bacteria, there are other methods. For instance, citrate is a weak iron chelator, and ferric citrate complexes can be imported through the ferric citrate transporter, or *fec*, system (21, 22). Studies investigating E. coli strains that cause bovine mastitis (mammary pathogenic E. coli [MPEC]) discovered that *fec* was a major pathogenic determinate of these strains (23, 24): in 62 MPEC strains, ~98% carried *fec* system genes (23). The high citrate levels in milk (~10 mM) provide a pool of ferric citrate for these bacterial strains to use as an iron source via the *fec* system, allowing MPEC to grow in milk and induce mastitis (23, 24).

Overall, little has been done to define the role of the *fec* system in the context of pathogenesis. However, there is a substantial body of work defining its regulation and mechanism of action. The *fec* system is composed of two operons: *fecIR* and *fecABCDE* (25–27). *fecIR* is Fur regulated and expressed under iron-limiting conditions, while *fecABCDE* is specifically transcribed via FecI, an alternative sigma factor, when ferric citrate is present (22, 28). FecA is a TonB-dependent outer membrane receptor, and FecBCDE comprise an ABC transporter (25, 26, 29).

In this study, we investigated the redundancy of iron acquisition systems in a collection of UPEC strains that caused symptomatic UTI in healthy, college-age women (30, 31). One of these clinical isolates, HM7, lacked the high diversity in iron acquisition systems characteristic of most UPEC strains. While this strain encoded free iron transporters (Feo and Efe), its only system to acquire iron from a sequestered source is through a single siderophore, enterobactin. While this strain lacks the diversity of virulence-associated methods of iron acquisition and has no clear mechanism to prevent Lcn2 from inactivating enterobactin, it is clearly pathogenic as it was isolated from a young woman with cystitis and was present in the urine at ≥10^5^ CFU/mL. In this study, we sought to determine how this novel strain acquires iron from the host. First, we empirically demonstrated that Lcn2-susceptible enterobactin is the sole siderophore produced by HM7. Using RNA-sequencing (RNA-seq), we found the ferric citrate uptake system highly upregulated under iron limitation and discovered that the *fec* system is enriched in UPEC isolates compared to fecal strains. Furthermore, HM7 can use ferric citrate as an iron source through the *fec* system and enterobactin *in vitro*, and the *fec* system is a fitness factor *in vivo*. Our study characterizes ferric citrate uptake as a UPEC virulence factor, adding another iron-scavenging mechanism that UPEC uses to survive within the urinary tract.

## RESULTS

### Clinical UPEC isolate HM7 lacks all but one of the iron acquisition systems associated with UPEC.

**FIG 1:**
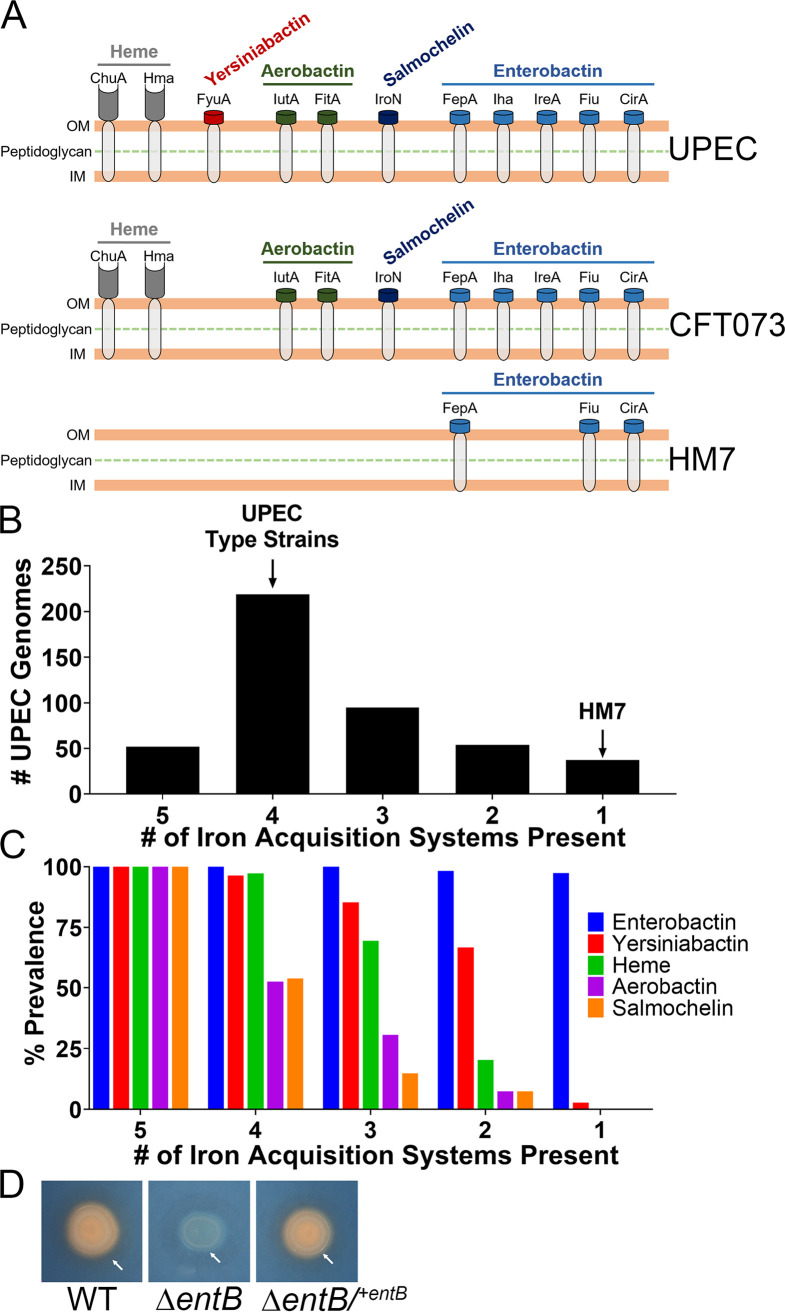
Clinical UPEC isolate HM7 encodes a single siderophore. (A) Models of siderophores, siderophore uptake receptors, and heme receptors in UPEC. “UPEC” indicates all known systems that have been found in UPEC, while “CFT073” and “HM7” illustrate the systems in each of these indicated strains. (B) Number of iron acquisition systems present in a cohort of 457 UPEC strains on the bioinformatics resource PATRIC. The five systems are composed of heme uptake (ChuA or Hma) and four siderophores (enterobactin, salmochelin, aerobactin, and yersiniabactin). Presence was determined by ≥80% protein identity and coverage of select genes for each system: heme uptake (*chuA* or *hma*), enterobactin (*entB*), salmochelin (*iroB*), aerobactin (*iucA*), and yersiniabactin (*irp1*). Sequence was used from strain CFT073 for all genes, with the exception of *irp1*, where strain 536 was used. Genes selected for siderophores are all involved in biosynthesis. Eleven percent of strains have five systems, 48% of strains have four, 21% of strains have three, 12% of strains have two, and 8% of strains have one. (C) The prevalence of each iron acquisition system within each subdivided group. (D) Siderophore production assayed through growth on CAS agar. Five microliters of overnight LB cultures was spotted onto CAS agar, and the cultures were grown overnight at 37°C. A change from blue to orange indicates siderophore activity. White arrows indicate the colonies in all three strains, and the orange halos in the WT and complemented strains are due to diffusion of secreted siderophore.

Roughly, there are up to five systems that UPEC strains use to acquire iron from the host (Fig. 1A). Most UPEC strains encode four of the five systems, including the three major UPEC type strains (CFT073, UTI89, and 536) (Fig. 1A and B). However, recent clinical isolate HM7 (30) encodes a single system, enterobactin, (Fig. 1A and B). After analyzing 457 publicly available UPEC strains on the bioinformatics resource PATRIC (32) (see Table S1 in the supplemental material), 37 strains shared the same profile as HM7, indicating that HM7 potentially represents a previously unrecognized subset of UPEC strains (Fig. 1B). We went on to assess the prevalence of each iron acquisition system within these subdivided groups and found a clear hierarchy within these different systems. Enterobactin was present in almost every single UPEC isolate, followed in prevalence by yersiniabactin, heme acquisition, aerobactin, and finally salmochelin (Fig. 1C).

10.1128/mbio.01035-22.9 TABLE S1 Strain list of UPEC, EHEC, and fecal E. coli isolates used in the UPEC, EHEC, and fecal cohorts. Download Table S1, XLSX file, 0.03 MB. Copyright © 2022 Frick-Cheng et al. 2022 Frick-Cheng et al. https://creativecommons.org/licenses/by/4.0/ This content is distributed under the terms of the Creative Commons Attribution 4.0 International license.

### HM7 encodes a single siderophore.

It was not clear how HM7 acquired iron and survived in the host since the innate immune protein Lcn2 renders enterobactin inaccessible to bacteria (20). Therefore, we hypothesized that HM7 encodes a novel siderophore. To test this, we deleted the gene *entB* (Δ*entB*), which is sufficient to disrupt enterobactin production (18), and tested siderophore production by culturing the deletion mutant on Chrome Azurol S (CAS) agar, a colorimetric iron chelation assay (33). A color change from blue to orange on the plate indicates iron chelation, which is observed on the colony itself as well as a halo around the colony due to diffusion of secreted siderophores. The wild-type (WT) strain showed robust siderophore activity that was absent in the Δ*entB* mutant but was subsequently restored by genetic complementation (Δ*entB/^+entB^*) (Fig. 1D). These results indicate that enterobactin is the sole siderophore system in HM7.

### Ferric citrate uptake is significantly upregulated during iron restriction.

HM7 did not make a novel siderophore; therefore, we predicted that it might utilize a previously undiscovered or understudied iron acquisition system. To identify a list of candidate genes, we used RNA-seq to determine the iron regulon of HM7. We added increasing amounts of the iron-specific chelator 2,2′-dipyridyl (Dip) to minimal M9 medium supplemented with 0.4% glucose to define an iron-restricted condition. The addition of 150 μM Dip to the base medium was sufficient to modestly limit growth due to iron restriction without introducing a severe growth defect. M9 supplemented with 36 μM FeCl_3_ (34, 35) comprised the iron-replete condition (Fig. S1A). We confirmed these conditions reflected iron-restricted and iron-replete conditions through quantitative reverse transcription-PCR (qRT-PCR); the iron-regulated gene *entF* was significantly and highly upregulated under the iron-restricted condition (Fig. S1B).

10.1128/mbio.01035-22.2 FIG S1 (A) Growth of WT HM7 in M9 medium with 0.4% glucose as the sole carbon source (M9), as well as supplemented with 36 μM FeCl_3_ or increasing amounts of the iron chelator 2,2′-dipyridyl (Dip). WT HM7 was cultured overnight in M9 and then subcultured 1:100 into 3 mL medium in culture tubes and grown at 37°C with aeration. OD_600_ was measured on a plate reader for 8 h, taking a reading every hour, and then another reading was taken at 24 h. Results are an average from three to four biological replicates; error bars represent ±SEM. (B) Gene expression of *entF* in WT HM7 in M9 medium supplemented with 150 μM Dip compared to M9 supplemented with 36 μM FeCl_3_. Gene expression was assayed through qRT-PCR. Bars are the average from three biological replicates, dots are the values from each biological replicate, error bars are ±SEM, and asterisks indicate significant upregulation, determined by one-sample *t* test: **, *P* < 0.01. Download FIG S1, TIF file, 0.3 MB. Copyright © 2022 Frick-Cheng et al. 2022 Frick-Cheng et al. https://creativecommons.org/licenses/by/4.0/ This content is distributed under the terms of the Creative Commons Attribution 4.0 International license.

**FIG 2:**
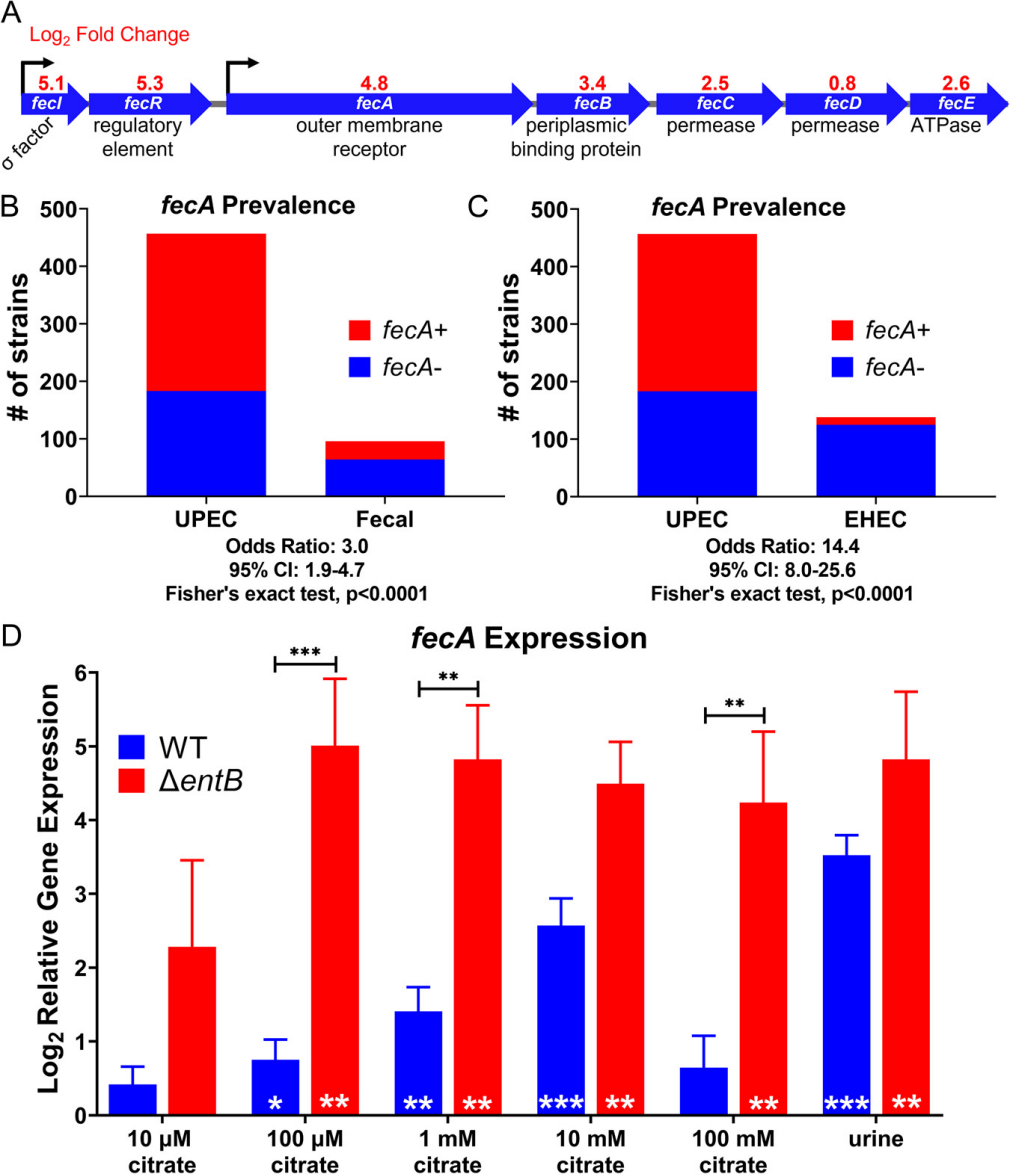
Ferric citrate uptake is a potential iron acquisition system in UPEC. (A) RNA-seq revealed the ferric citrate uptake system (*fecABCDE* and *fecIR*) is upregulated in WT HM7 under iron limitation (M9 supplemented with 36 μM FeCl_3_ versus M9 with 150 μM 2,2′-dipyridyl). (B) *fecA* is enriched in UPEC strains compared to E. coli fecal isolates. 457 UPEC strains and 96 fecal strains were analyzed; the presence of *fecA* was determined by ≥80% protein identity and coverage. (C) *fecA* is enriched in UPEC strains compared to enterohemorrhagic E. coli (EHEC). 457 UPEC strains and 139 EHEC strains were analyzed. (D) Gene expression of *fecA* in HM7 in either M9 medium with 0.4% glucose supplemented with increasing amounts of citrate or in pooled human urine. Gene expression was assayed through qRT-PCR. Bars are the mean from six biological replicates, and error bars are ±standard error of the mean (SEM). Black asterisks compare gene expression between WT and the Δ*entB* mutant using mixed-effects analysis with Sidak’s multiple test correction: **, *P* < 0.01; ***, *P* < 0.001. White asterisks indicate significant upregulation relative to M9 medium without citrate, determined by one-sample *t* test: *, *P* < 0.05; **, *P* < 0.01; ***, *P* < 0.001.

HM7 was cultured to mid-log phase under these conditions in biological triplicate, its RNA was isolated and sequenced. A total of 368 genes were significantly downregulated under the iron-depleted condition (Table S2), while 393 genes were significantly upregulated (Table 1; Table S2). As expected, we observed that the genes for enterobactin biosynthesis and uptake, as well as genes associated with iron starvation (*nrdEFH*) (36) and Fur-regulated genes (*fhuEF* and *fiu*) (37), were upregulated (Table 1). Two transport systems related to iron were significantly upregulated. One was encoded by *mntH* and is a system that takes up both Mn^2+^ and Fe^2+^, although with a preference for Mn^2+^ (38). The other system was ferric citrate uptake, which is composed of two operons, *fecIR*, encoding the system’s regulatory element and sigma factor, and *fecABCDE*, encoding the outer membrane receptor and transport elements (Fig. 2A). Interestingly, *fecD* was not significantly upregulated. Unlike *mntH*, the *fec* system takes up Fe^3+^, which is dominant form of iron in the urinary tract. Furthermore, citrate is present is extremely high levels in the urinary tract: normal levels in healthy individuals vary from 1.7 to 6.6 mM (39). Given that MPEC uses ferric citrate in bovine milk, and the citrate concentration in milk (~10 mM) is comparable to the concentration in urine, we hypothesized that UPEC is using a similar mechanism in the urinary tract.

**TABLE 1:** Top 50 significantly upregulated genes under iron limitation

Gene	Product description	Log_2_ FC^*a*^	Locus tag
*adhP*	Alcohol dehydrogenase	4.6	b1478
*aroF*	Phospho-2-dehydro-3-deoxyheptonate aldolase	4.1	EICMKPFN_03556
*bioA*	Adenosylmethionine-8-amino-7-oxononanoate aminotransferase	4.0	b0774
*cirA*	Outer membrane receptor for ferrienterochelin and colicins	5.0	b2155
*EICMKPFN_01803*	Phosphate starvation-inducible protein	3.9	EICMKPFN_01803
*EICMKPFN_02077*	Hypothetical protein	4.2	EICMKPFN_02077
*EICMKPFN_02251*	Glyceraldehyde-3-phosphate dehydrogenase	4.7	EICMKPFN_02251
*EICMKPFN_02252*	Glyceraldehyde-3-phosphate dehydrogenase	4.5	EICMKPFN_02252
*EICMKPFN_03110*	Colicin I receptor	4.5	EICMKPFN_03110
*entA*	2,3-Dihydro-2,3-dihydroxybenzoate dehydrogenase	5.4	b0596
*entB*	Enterobactin synthase component B	5.8	b0595
*entC*	Isochorismate synthase EntC	4.7	b0593
*entD*	Enterobactin synthetase component D	5.0	b0583
*entE*	2,3-Dihydroxybenzoate-AMP ligase	5.1	b0594
*entF*	Enterobactin synthetase component F	6.9	b0586
*entH*	Proofreading thioesterase in enterobactin biosynthesis	5.0	b0597
*fecA*	Ferric citrate outer membrane transporter	4.8	b4291
*fecI*	RNA polymerase σ^19^ factor	5.2	b4293
*fecR*	Regulator for *fec* operon	5.3	b4292
*fepA*	Ferric enterobactin receptor	5.4	b0584
*fes*	Fe^+3^-enterobactin esterase	5.3	b0585
*fhuE*	Outer membrane receptor for ferric coprogen and ferric-rhodotorulic acid	5.4	b1102
*fhuF*	Ferric iron reductase protein	4.6	b4367
*fiu*	Catecholate siderophore receptor	4.4	b0805
*gabP*	4-Aminobutyrate:H(+) symporter	4.7	b2663
*gadA*	Glutamate decarboxylase A	5.1	b3517
*gadB*	Glutamate decarboxylase B	5.2	b1493
*gadC*	l-Glutamate:4-aminobutyrate antiporter	5.4	b1492
*gcd*	Quinoprotein glucose dehydrogenase	4.2	b0124
*hchA*	d-Lactate dehydratase	4.5	b1967
*mntH*	Manganese transport protein	5.6	b2392
*nrdE*	Ribonucleoside-diphosphate reductase 2 subunit alpha	6.1	b2675
*nrdF*	Ribonucleoside-diphosphate reductase 2 subunit beta	6.8	b2676
*nrdH*	Glutaredoxin-like protein	6.3	b2673
*nrdI*	Protein involved in ribonucleotide reduction	5.9	b2674
*phoH*	Phosphate starvation-inducible protein	4.1	b1020
*sufC*	Fe-S cluster assembly ATP-binding protein	4.0	b1682
*sufD*	Fe-S cluster assembly protein	4.1	b1681
*sufE*	Cysteine desulfuration protein	3.8	b1679
*sufS*	Selenocysteine lyase	4.1	b1680
*tyrA*	Chorismate mutase	3.7	b2600
*ybdZ*	Enterobactin biosynthesis protein	6.1	b4511
*ybgS*	Uncharacterized protein	4.3	b0753
*ybiX*	PKHD-type hydroxylase	3.7	b0804
*yciG*	Uncharacterized protein	4.5	b1259
*yddM*	Putative DNA-binding transcriptional regulator	4.6	b1477
*ydiE*	Uncharacterized protein	4.2	b1705
*yjjZ*	Uncharacterized protein	7.3	b4567
*yncE*	PQQ-like domain-containing protein	5.0	b1452
*yohC*	Putative inner membrane protein	3.7	b2135

a FC, fold change.

10.1128/mbio.01035-22.10 TABLE S2 All genes significantly (adjusted *P* value of <0.05) differentially regulated (log_2_ FC of <1 or >1) in UPEC isolate HM7 under iron limitation. Download Table S2, XLSX file, 0.04 MB. Copyright © 2022 Frick-Cheng et al. 2022 Frick-Cheng et al. https://creativecommons.org/licenses/by/4.0/ This content is distributed under the terms of the Creative Commons Attribution 4.0 International license.

### *fecA* is highly prevalent in UPEC strains.

We wanted to establish the prevalence of the *fec* system in UPEC strains, since three UPEC type strains, CFT073, UTI89, and 536, lack the *fec* system. When we interrogated the cohort of 457 UPEC strains, we found that ~61% of them carried the outer membrane receptor gene *fecA*, compared to only ~33% in 96 fecal E. coli isolates (Fig. 2B). This is a significant association, with an odds ratio of 3.0, supporting the hypothesis of ferric citrate uptake as a UPEC virulence factor. Within UPEC, there was only a subtle, but not significant, enrichment of *fecA* in strains with a sole iron acquisition system (“HM7-like”) to more traditional UPEC strains with four virulence-associated iron acquisition systems (Fig. S2). However, the enrichment of *fecA* is even stronger comparing UPEC to enterohemorrhagic E. coli (EHEC), with an odds ratio of 14.4 (Fig. 2C); only ~9% of 138 EHEC strains carry *fecA*.

10.1128/mbio.01035-22.3 FIG S2 *fecA* is enriched in UPEC strains with a single traditional iron acquisition system (“HM7-like” [37 strains]) compared to strains with four traditional iron acquisition systems (219 strains). Download FIG S2, TIF file, 0.2 MB. Copyright © 2022 Frick-Cheng et al. 2022 Frick-Cheng et al. https://creativecommons.org/licenses/by/4.0/ This content is distributed under the terms of the Creative Commons Attribution 4.0 International license.

### *fecA* is responsive to physiologically relevant levels of citrate.

HM7 is a mostly uncharacterized clinical isolate; therefore, we wanted to determine if the *fec* system is fully functional and responsive to citrate at physiologically relevant levels. We cultured WT HM7 in M9 with glucose as a carbon source and supplemented with concentrations of citrate ranging from 10 μM to 100 mM, which encompasses urinary citrate levels in a healthy population (39). We quantified *fecA* gene expression compared to that in M9 without citrate. We observed significant upregulation at 100 μM, 1 mM, and 10 mM citrate (Fig. 2D), and importantly, some of the strongest upregulation occurred at physiologically relevant concentrations (1 mM and 10 mM citrate). We also tested *fecA* expression in *ex vivo* urine pooled from healthy female volunteers and compared it to expression in M9 without citrate. *fecA* was significantly upregulated (Fig. 2D) in this physiologically relevant medium.

### *fecA* is more highly upregulated in the absence of enterobactin.

We hypothesized that HM7 relies on ferric citrate uptake to acquire iron in the absence of enterobactin, indicating the strain can use ferric citrate as an alternative iron source. To test this hypothesis, we repeated the citrate sensitivity experiments using the Δ*entB* mutant. Significant upregulation at 100 μM, 1 mM, and 10 mM citrate was recapitulated in the mutant strain (Fig. 2D). Furthermore, at 100 μM, 1 mM, and 100 mM citrate, the Δ*entB* mutant had significantly higher expression of *fecA* than the WT. *fecA* expression was also highly upregulated in the Δ*entB* mutant in pooled human urine. Interestingly, while expression of *fecA* dropped at 100 mM citrate in the WT, it remained highly elevated in the Δ*entB* mutant, indicating that perhaps enterobactin is the preferred mechanism for iron acquisition, but in its absence, the *fec* system can be utilized. These results support the hypothesis that HM7 is using ferric citrate as an iron source, especially in the absence of Fe^3+^ uptake by siderophores.

### HM7 uses ferric citrate as an iron source through the the *fec* system or enterobactin.

**FIG 3:**
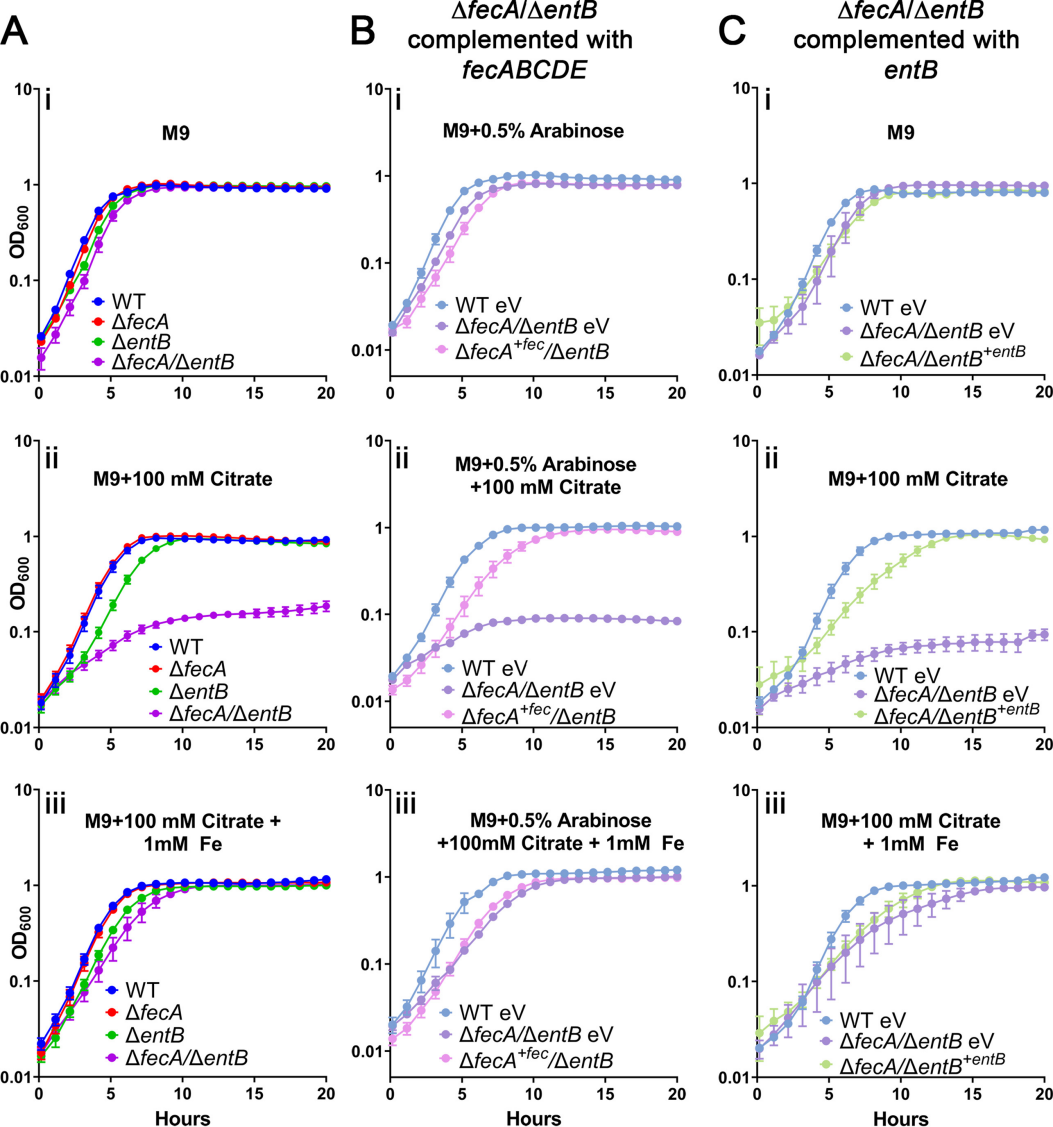
HM7 uses ferric citrate as an iron source through the *fec* system and enterobactin. Shown is growth in M9 medium (i), M9 medium supplemented with 100 mM citrate (ii), and M9 medium supplemented with both 100 mM citrate and 1 mM FeCl_3_ (iii) of (A) WT HM7, the Δ*fecA* and Δ*entB* single mutants, and the Δ*fecA*/Δ*entB* double mutant and (B) WT HM7 expressing empty pBAD vector (WT eV), the Δ*fecA*/Δ*entB* mutant expressing empty pBAD vector (Δ*fecA/*Δ*entB* eV), and the Δ*fecA*/Δ*entB* mutant complemented with *fecABCDE (*Δ*fecA^+fec^*/Δ*entB*). All media under these conditions were supplemented with 0.5% arabinose to induce expression. (C) WT HM7 expressing empty pGEN vector (WT eV), the Δ*fecA*/Δ*entB* mutant expressing empty pGEN vector (Δ*fecA/*Δ*entB* eV), and the Δ*fecA*/Δ*entB* mutant complemented with *entB* under the control of its native promoter (Δ*fecA/*Δ*entB^+entB^*). A 0.4% concentration of glucose was used as the sole carbon source under all conditions. Growth curves show averages from three to five biological replicates; error bars are SEM.

To determine if HM7 can use ferric citrate as an iron source, we added 100 mM citrate to M9 to sequester most of the iron within citrate. The bacteria have two ways to acquire iron: either enterobactin will chelate iron from ferric citrate, or the *fec* system will import ferric citrate. To nullify ferric citrate uptake, we deleted the outer membrane receptor gene *fecA* (Δ*fecA*). We also constructed a double mutant (Δ*fecA*/Δ*entB*). With these assumptions, only the double mutant would have a growth defect at high citrate concentrations, since the Δ*fecA* mutant could still utilize enterobactin, and the Δ*entB* mutant could still utilize the *fec* system. As expected, only the Δ*fecA*/Δ*entB* mutant had a profound growth defect with the addition of 100 mM citrate (Fig. 3Aii), while none of the mutants had a growth defect in LB or M9 alone (Fig. 3Ai; Fig. S3A). This is an iron-specific defect since chemical complementation with 1 mM FeCl_3_ rescued the growth of the double mutant (Fig. 3Aiii).

10.1128/mbio.01035-22.4 FIG S3 Growth of WT HM7, and the Δ*fecA*, Δ*entB*, and Δ*fecA*/Δ*entB* mutants in LB (A) or *ex vivo* urine pooled from healthy female volunteers (B). Results are an average from four to five biological replicates; bars represent ±SEM. Download FIG S3, TIF file, 0.4 MB. Copyright © 2022 Frick-Cheng et al. 2022 Frick-Cheng et al. https://creativecommons.org/licenses/by/4.0/ This content is distributed under the terms of the Creative Commons Attribution 4.0 International license.

To establish that HM7 could specifically use the *fec* system to acquire iron via ferric citrate, we took a genetic approach, complementing the Δ*fecA*/Δ*entB* double mutant with each single system. Unsurprisingly, growth of the Δ*fecA*/Δ*entB* double mutant was rescued by genetic complementation with *entB* (Fig. 3C). However, *fecABCDE* was also sufficient to rescue growth (Fig. 3B). *fecA* was not sufficient to rescue growth, indicating that the Δ*fecA* mutant is a polar mutation, although that does not change the interpretation of our results.

### Ferric citrate uptake is an *in vitro* fitness factor when HM7 cannot utilize enterobactin.

Not only is *fecA* associated with UPEC strains (Fig. 2B and C), but the *fec* system allows HM7 to use ferric citrate as an iron source (Fig. 3). We hypothesized that *fec* system could provide UPEC with a competitive advantage. Initially, we assessed the growth of the WT and Δ*fecA*, Δ*entB*, and Δ*fecA*/Δ*entB* mutant strains in pooled human urine (Fig. S3B). Surprisingly there seemed to be no significant growth defect in any of these mutants compared to the WT. Therefore, we turned to a more sensitive assay to assess the advantage *fec* could provide and performed competition experiments in pooled human urine.

We competed WT against the Δ*fecA* mutant and observed no competitive disadvantage of the mutant strain compared to the WT (Fig. S4). Both strains could still use enterobactin; perhaps the siderophore is the preferred mechanism to acquire iron. This was confirmed as both the Δ*entB* and Δ*fecA*/Δ*entB* strains had a significant disadvantage compared to the WT, but the double mutant did not have a larger defect than the Δ*entB* mutant (Fig. S4). These results indicate that enterobactin contributes to the survival of HM7, but also masks the role of *fec.*

10.1128/mbio.01035-22.5 FIG S4 Indicated strains were competed in pooled human urine. Black asterisks compare log_10_ CIs between indicated strains using ordinary one-way ANOVA with Sidak’s multiple-test correction: *, *P* < 0.05; ****, *P* < 0.0001. Red asterisks indicate a significant competitive disadvantage, determined by one-sample *t* test: *, *P* < 0.05; **, *P* < 0.005. Download FIG S4, TIF file, 0.3 MB. Copyright © 2022 Frick-Cheng et al. 2022 Frick-Cheng et al. https://creativecommons.org/licenses/by/4.0/ This content is distributed under the terms of the Creative Commons Attribution 4.0 International license.

**FIG 4:**
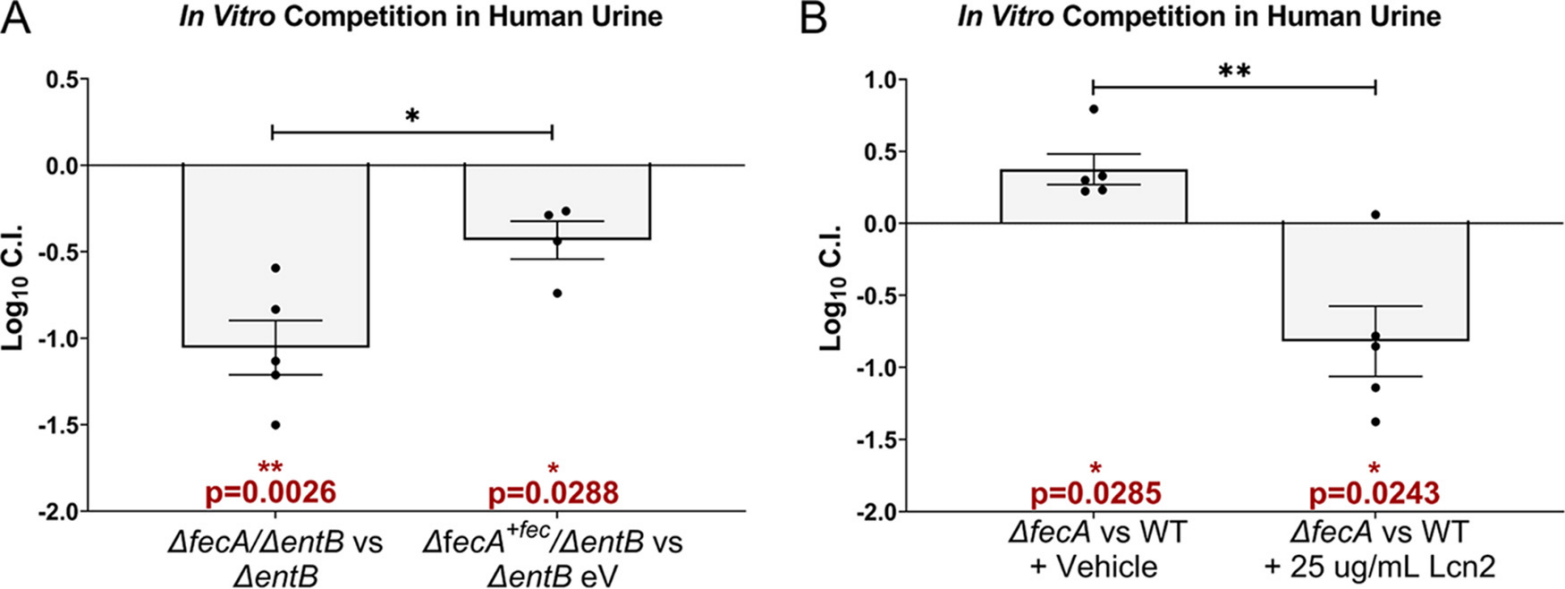
Ferric citrate uptake is an *in vitro* fitness factor in the absence of enterobactin. *In vitro* fitness of strains or conditions was determined in *ex vivo* pooled human urine. All strains were inoculated in a 1:1 ratio and grown for 24 h at 37°C with aeration, and their log_10_ competitive index (CI) was determined. A log_10_ CI of <0 indicates the first listed strain was outcompeted by the second. (A) The Δ*entB* mutant expressing an empty vector (Δ*entB* eV) and the Δ*fecA*/Δ*entB* double mutant with the *fec* operon complemented in *trans* (Δ*fecA^+fec^/*Δ*entB*) were competed in urine supplemented with 0.5% arabinose and ampicillin (100 μg/mL). (B) WT HM7 was competed with the Δ*fecA* mutant, and the urine was supplemented with either recombinant human lipocalin (Lcn2) or an equal volume of vehicle (25% glycerol). Red asterisks indicate a significant competitive disadvantage, determined by one-sample *t* test: *, *P* < 0.05; **, *P* < 0.005. Black asterisks compare log_10_ CIs between indicated strains or conditions using an unpaired *t* test: *, *P* < 0.05; **, *P* < 0.005. Bars indicate the mean; error bars are ±SEM. Each dot represents an independent experiment.

To dissect the precise contribution of the *fec* system in the absence of enterobactin, we competed the Δ*entB* mutant with the Δ*fecA*/Δ*entB* double mutant and observed a loss in fitness of the double mutant (Fig. 4A). This defect is largely specific to the *fec* system since complementing the double mutant with *fecABCDE* was sufficient to partially rescue the defect (Fig. 4A), indicating that the fitness advantage provided by the *fec* system is contingent on a lack of enterobactin. However, this condition is highly relevant due to the high levels of the enterobactin-binding protein Lcn2 in the urinary tract during UTI (40–42). Therefore, to mimic the host’s infectious environment, we added recombinant Lcn2 to these competitions. We determined that 25 μg/mL of Lcn2 was sufficient to inhibit HM7 growth in an iron-limited environment (Fig. S5) and then supplemented pooled human urine with that amount and competed the WT and the Δ*fecA* mutant. With the addition of Lcn2, the Δ*fecA* mutant now had a significant competitive disadvantage (Fig. 4B). This provides further evidence that in the absence or inhibition of enterobactin, the *fec* system is a fitness factor.

10.1128/mbio.01035-22.6 FIG S5 Growth of WT HM7 supplemented with recombinant human lipocalin (Lcn2). WT HM7 was grown in an iron-starved state (M9 medium supplemented with 150 μM Dip) with increasing amounts of Lcn2. An equal volume of the vehicle (vh [25% glycerol]) for each amount was added as a control. Results are an average from two biological replicates; bars represent ±SEM. Download FIG S5, TIF file, 0.3 MB. Copyright © 2022 Frick-Cheng et al. 2022 Frick-Cheng et al. https://creativecommons.org/licenses/by/4.0/ This content is distributed under the terms of the Creative Commons Attribution 4.0 International license.

### Ferric citrate uptake is an *in vivo* fitness factor.

**FIG 5:**
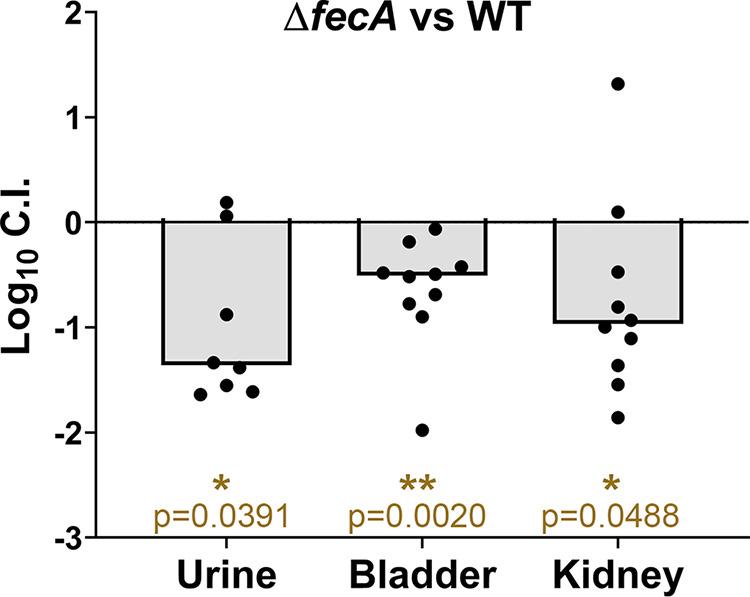
Ferric citrate uptake is an *in vivo* fitness factor. WT HM7 and the Δ*fecA* mutant were combined in a 1:1 ratio and transurethrally inoculated into CBA/J mice. Competitive indices were calculated 48 h postinfection. Symbols are individual animals; bars are the median. Significance was determined with Wilcoxon’s signed-rank test: *, *P* < 0.05; **, *P* < 0.005.

Finally, we wanted to determine if the *fec* system was an *in vivo* fitness factor. Using the ascending UTI mouse model, we coinfected female CBA/J mice with WT and the Δ*fecA* mutant, allowed the infection to progress for 48 h, and harvested the urine, bladder, and kidneys to calculate the log_10_ competitive index (CI). The Δ*fecA* mutant had a significant disadvantage in all three organ sites (Fig. 5), definitively defining it as a fitness factor in UPEC.

We hypothesized that the Δ*fecA* mutant had a defect *in vivo* due to the presence of Lcn2, as seen in the *in vitro* competitions supplemented with Lcn2 (Fig. 4B). Lcn2 is highly elevated in the bladders and kidneys of mice infected with WT HM7 (Fig. S6A and B). Lcn2 levels correlated with increased CFU burden in the kidneys, where Lcn2 is produced (43) (Fig. S6C). To determine if Lcn2 was responsible for the competitive disadvantage of the Δ*fecA* mutant, we repeated the competition experiments with Lcn2 knockout (*Lcn2*^−/−^) mice. However, the *Lcn2*^−/−^ mice are in a different genetic background, C57BL/6, rather than CBA/J, so we repeated the competition in the WT (C57BL/6) mouse background as well. While there was a subtle difference in the log_10_ CIs of the bladders between WT and Lcn2^−/−^ mice that was trending toward significance, the Δ*fecA* mutant no longer had a disadvantage compared to the WT in the C57BL/6 background (Fig. S7). This discrepancy in results reflects the differences between mouse strains. Overall, we conclude that ferric citrate uptake through the *fec* system is a bona fide fitness factor in UPEC strain HM7, allowing it to acquire iron from the host in a manner not inhibited by Lcn2.

10.1128/mbio.01035-22.7 FIG S6 Quantification of lipocalin (Lcn2) production during murine infection. CBA/J mice were infected either with WT HM7 or mock infected with PBS. Lcn2 levels were quantified via ELISA in the (A) bladder and (B) kidneys. (C) Lcn2 levels were plotted against CFU burden of mice infected with HM7. The Pearson correlation coefficient (*r*) for the bladder is displayed in blue, and that for kidneys is displayed in red. Dots indicate individual mice; bars are the median. Significance was determined via Mann-Whitney test: *, *P* < 0.05. Download FIG S6, TIF file, 0.2 MB. Copyright © 2022 Frick-Cheng et al. 2022 Frick-Cheng et al. https://creativecommons.org/licenses/by/4.0/ This content is distributed under the terms of the Creative Commons Attribution 4.0 International license.

10.1128/mbio.01035-22.8 FIG S7 WT HM7 competed with the Δ*fecA* mutant in WT C57BL/6 mice (WT) and lipocalin null (*Lcn2*^−/−^) mice. Competitive indices were calculated 48 h postinfection. Historically, C57BL/6 mice have poor kidney colonization: only 3/10 WT mice and 4/10 *Lcn2*^−/−^ mice had detectable CFU in the kidney. Dots are individual animals, and bars are the median. Log_10_ CIs were compared using the Mann-Whitney test. Download FIG S7, TIF file, 0.1 MB. Copyright © 2022 Frick-Cheng et al. 2022 Frick-Cheng et al. https://creativecommons.org/licenses/by/4.0/ This content is distributed under the terms of the Creative Commons Attribution 4.0 International license.

## DISCUSSION

Iron acquisition is an essential virulence factor in UPEC, because most iron in the host is sequestered. Subsequently, UPEC relies on specific iron acquisition systems such as siderophores or heme receptors to scavenge iron from otherwise inaccessible sources, and these systems are essential for UPEC pathogenesis (10, 44, 45). Our work shows there is another understudied and overlooked iron acquisition system that enhances UPEC pathogenesis, ferric citrate uptake, encoded by the *fec* system.

Our study focuses on a recent clinical UPEC isolate, HM7. This strain encodes the synthesis pathway for a sole siderophore, enterobactin, and lacks the diversity in iron acquisition systems normally observed in UPEC strains. We hypothesized that HM7 was employing another method to acquire iron from the host and used RNA-seq to define its iron regulon. Under iron-limiting conditions, we found almost every component of ferric citrate uptake (*fecABCE* and *fecIR*) was highly and significantly upregulated (Table 1 and Fig. 2A; see Table S2 in the supplemental material). Interestingly, *fecD* was not highly upregulated. While the rest of the genes in the system had log_2_ fold change (FC) values ranging from 2.6 to 5.1, *fecD* had a log_2_ FC of 0.8, and unlike the rest, this change was not significant. This is intriguing as *fecD* is the second-to-last gene in the operon, and yet the gene after it, *fecE*, is significantly and highly upregulated. *fecD* and *fecC* encode the permeases of the transport system that form a channel in the inner membrane of the bacterium (26, 46). Permeases can form homodimers or heterodimers, and it is tempting to speculate the modest upregulation of *fecD* indicates that there is a preference for FecC homodimers as opposed to FecC/FecD heterodimers. Potentially, *fecE* uses an alternative start site, explaining its higher expression levels. *fecE* encodes the ATPase of this system, which is essential for activity of this ABC transporter. Precisely defining this mechanism will require future studies.

We uncovered that the *fec* system is enriched in UPEC strains compared to fecal strains (odds ratio of 3.0) (Fig. 2B). Given how common the *fec* system is within UPEC and MPEC, we wondered if it could be a virulence factor in other pathogenic E. coli strains. However, we discovered that the *fec* system had an even stronger odds ratio (14.4) in UPEC than in EHEC (Fig. 2C). Together, these results indicate that enrichment of the *fec* system is potentially niche specific, as it is associated with E. coli strains where there is abundant citrate (urinary tract and milk). In contrast, EHEC colonizes the gut, where most iron would be ferrous due to anoxic conditions, as opposed to the ferric form required to complex with citrate, thus possibly reducing the utility of the *fec* system.

However, another important infection niche that E. coli can infect is the bloodstream, and perhaps these strains could also utilize *fec* to acquire iron. The citrate levels in plasma vary from 100 to 150 μM, and while these levels are lower than those in urine or milk, they are still sufficient for robust upregulation of *fecA* (Fig. 2D). In fact, a recent study exploring conjugative plasmids in pathogenic E. coli found a plasmid that carried the *fec* system conferred a modest *in vivo* competitive advantage during bacteremia (47). This was also tested in the UTI model, and when this plasmid was conjugated into a different E. coli strain, loss of *fec* resulted in an extremely mild reduction in fitness (log_10_ CIs of approximately −0.1 in the bladder and approximately −0.2 in the kidneys). However, this result could not be recapitulated in its parent strain. Other iron acquisition systems in these strains were not defined and could explain the divergence of results, demonstrating how diversity of iron acquisition systems can mask the contributions of specific systems.

*In vitro* competition in pooled human urine showed the *fec* system provides a competitive advantage contingent on the absence of enterobactin (Fig. 4). The *fec* system had an advantage either when both strains lacked *entB* or when Lcn2 was present at levels sufficient to inhibit enterobactin (Fig. 4B). Lcn2 is present at high levels in the urinary tract during infection (40–42); therefore, these *in vitro* competitions with the addition of Lcn2 are likely a closer representation of UTI.

The gene expression profile of UPEC during CBA/J mouse infection closely mimics the UPEC transcriptome during human infection (48). Therefore, we are reasonably confident that the results from the mouse model are relevant to human infection. When WT HM7 was competed against the Δ*fecA* mutant, the mutant had a disadvantage in the urine, bladder, and kidneys (Fig. 5). While this result is different than the *in vitro* competition in human urine alone, it aligns with the *in vitro* competitions supplemented with Lcn2, implying the *fec* system provides an advantage *in vivo* because HM7 is unable to use enterobactin, likely due to Lcn2. Mice infected with HM7 had robust production of Lcn2 in the bladder and kidneys (Fig. S6).

We attempted to confirm this hypothesis using *Lcn2*^−/−^ mice. If Lcn2 is essential for the competitive advantage of the *fec* system, that advantage should be abrogated in the knockout line. The *Lcn2*^−/−^ mice were in a C57BL/6 background; therefore, we retested WT HM7 and the Δ*fecA* mutant in WT C57BL/6 mice. Unfortunately, there was no loss in fitness in the Δ*fecA* mutant in WT C57BL/6 mice (Fig. S7). However, there are several genetic differences between these mouse lines (49) that could account for the divergence of results, and indeed the progression of UTI is quite varied between these murine lines (50). It is noteworthy that C57BL/6 mice are not commonly used; CBA (51) and C3H/HeN (52) are the mice most often used in experimental UTI. We observed this variation between mouse strains: of the 10 CBA/J mice we infected, 100% of them had kidney colonization, while only 35% of the 20 C57BL/6 mice had kidney colonization. While the contribution of Lcn2 to the mechanism of ferric citrate uptake via the *fec* system has not been definitively proven, it seems a promising explanation, or perhaps is one of several factors that could contribute.

**FIG 6:**
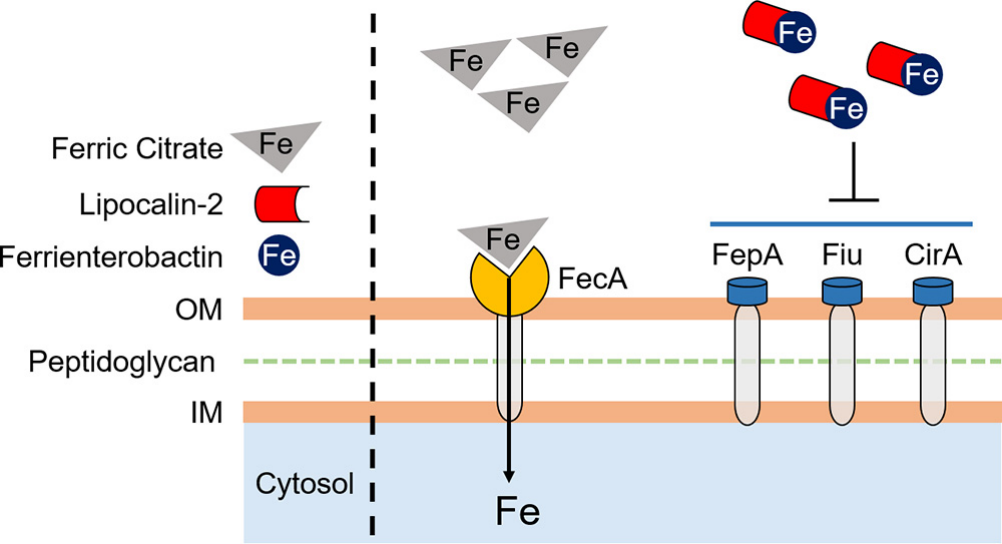
Model of UPEC utilization of ferric citrate. Clinical UPEC isolate HM7 encodes a biosynthetic pathway for a sole siderophore, enterobactin, as well as three enterobactin uptake receptors, FepA, Fiu, and CirA, to acquire iron during infection. In the presence of the immune protein Lcn2, enterobactin is rendered inaccessible to bacteria. In response, HM7 employs ferric citrate uptake through the *fec* system to acquire iron from the host.

In summary, we have uncovered an understudied mechanism by which UPEC acquires iron from the host via ferric citrate uptake. During UTI, Lcn2 is highly produced, blocking the usage of enterobactin. In response, UPEC uses the *fec* system to import ferric citrate present in the urinary tract as an iron source (Fig. 6). The *fec* system is highly prevalent in UPEC strains and is yet another instrument in its highly diverse arsenal to survive within the harsh environment of the urinary tract.

## MATERIALS AND METHODS

### Bacterial culture conditions, growth curves, mutant construction, and complementation.

Clinical UPEC isolate HM7 was routinely cultured at 37°C with aeration in LB, M9 medium supplemented with glucose, or filter-sterilized pooled human urine. Mutant and complemented strains were cultured with antibiotics. Mutants were constructed using lambda red mutagenesis and complementation vectors constructed with Gibson assembly. See Text S1 in the supplemental material for detailed description.

10.1128/mbio.01035-22.1 TEXT S1 All supplemental methods and expanded methods referenced in the main text. Download Text S1, DOCX file, 0.02 MB. Copyright © 2022 Frick-Cheng et al. 2022 Frick-Cheng et al. https://creativecommons.org/licenses/by/4.0/ This content is distributed under the terms of the Creative Commons Attribution 4.0 International license.

### Chrome Azurol S assay.

Chrome Azurol S (CAS) agar was prepared as described in reference 33. Strains were cultured overnight with aeration at 37°C in LB with appropriate antibiotics. Five microliters of the overnight culture was spotted onto the CAS-agar plate and incubated overnight at 37°C. The plates were imaged using Qcount software.

### RNA isolation and library preparation and sequencing.

E. coli HM7 was cultured overnight in M9 medium supplemented with 0.4% glucose, with shaking at 37°C. Overnight cultures were diluted 1:100 in M9 medium with 0.4% glucose supplemented with either 36 μM FeCl_3_ (Sigma) or 150 μM 2,2′-dipyridyl (Sigma) and grown to mid-log phase (OD_600_ of 0.4 to 0.6). Cultures were treated with bacterial RNAprotect (Qiagen) and harvested by centrifugation, and the pellets were stored at −80°C. This was performed in biological triplicate. RNA was isolated as defined in references 30 and 48. cDNA libraries were prepared using NEBNext Ultra II Directional RNA library prep kit and sequenced using an Illumina NextSeq-500 (paired-end, 38-bp read length). See Text S1 for detailed description.

### Genome assembly, RNA-seq data processing, and differential expression analysis.

Raw sequencing data were preprocessed using BBTools (38.18) (53). BBDuk was used to remove Illumina adapter sequences and to quality trim and filter the reads (minlength = 20, trimq = 14, maq = 20, maxns = 1). The HM7 genome was reassembled based on sequencing from reference 30 using the Flye long-read assembler (54) with the Trestle repeat resolve parameter on, and then the quality-controlled reads were aligned to the HM7 genome using BWA (0.7) (55). The resulting alignment files were filtered (mapping quality of >10) using samtools (1.11) (56), with counts for each feature generated using htseq-count (0.13.5) (57). Alignment details are shown in Table 2. A list of the strains and plasmids is provided in Table 3, and the primers used are listed in Table 4. Differential expression analysis was performed using R package DESeq2 (58).

**TABLE 2:** Numbers and percentages of mapped reads

Condition^*a*^	No. of reads		% of reads mapped
	Total	Mapped	
M9+Fe			
rep 1	37,012,780	36,963,041	99.9
rep 2	30,261,187	30,217,903	99.9
rep 3	31,443,502	31,408,578.5	99.9

M9+Dip			
rep 1	36,295,965	36,246,384.5	99.9
rep 2	36,201,265	36,139,361	99.8
rep 3	38,356,595	38,299,136	99.9

a rep, replicate.

**TABLE 3:** List of strains and plasmids used in this study

Strain or plasmid	Genotype/description	Reference or source
Strains		
HM7	WT cystitis-causing UPEC strain isolated from healthy young woman in 2012	
Δ*entB* mutant	HM7 *entB*::*kan* Kan^r^	This study
Δ*fecA* mutant	HM7 *fecA*::*cam* Cam^r^	This study
Δ*fecA*/Δ*entB* mutant	HM7 *fecA*::*cam entB*::*kan* Cam^r^ Kan^r^	This study

Plasmids		
pGEN eV	Low-copy-no., promoterless plasmid, Spec^r^	This study
pBAD eV	pBAD-*Myc*/His A, low-copy-no., arabinose-inducible plasmid, Amp^r^	Thermo Fisher
pGEN *entB*	*entB* with native promoter, cloned from HM7 via Gibson assembly, Spec^r^	This study
pBAD *fecABCDE*	*fec* operon (*fecABCDE*) cloned from HM7 inserted into MCS via Gibson assembly, Amp^r^	This study
pGEX-4T-3 LCN	Human lipocalin-2 glutathione *S*-transferase (GST) fusion protein, Amp^r^	

**TABLE 4:** Primers used in this study

Gene or plasmid	Sequence of^*a*^:	
	Forward primer	Reverse primer
*entB* ^*b*^	ATTCCAAAATTACAGGCTTACGCACTGCCGGAGTCGTGTAGGCTGGAGCTGCTTC	CACCTCGCGGGAGAGTAGCTTCCACCAGGCGTCGAATGGGAATTAGCCATGGTCC
*fecA* ^*b*^	GATGATGGGGAAGGTATGACGCCGTTACGCGTTTTTCGTAAAACAACACCGTGTAGGCTGGAGCTGCTTC	CCGGGCGTTAACACATCAGAACTTCAACGACCCCTGCATATACAGCGTGCATGGGAATTAGCCATGGTCC
P_native_*entB*^*c*^	CGGTACCAAGCTTCATATGCACAAATCAGCTTCCTGTTATTAATAAG	GAATAGCCATATCATCCTCCACAAAATG
*entB* ^*c*^	GGAGGATGATATGGCTATTCCAAAATTACAGG	TTCCTGCAGGGCATGCCCCGTTATTTCACCTCGCGGGAG
*fecABCDE* ^*c*^	GAGATCTGCAGCTGGTACCAATGACGCCGTTACGCGTTTTTCG	CCAAGCTTCGAATTCCCATACCTCATTAGGCACATCGGCCTGCS
pGEN^*c*^	CGGGGCATGCCCTGCAGG	GCATATGAAGCTTGGTACCGGGATCCGC
pBAD^*c*^	TATGGGAATTCGAAGCTTGGGCCCG	TGGTACCAGCTGCAGATCTCGAGC
*fecA* ^*d*^	CGGAAGGGCCGATCATAAA	TACCTGGAGCAAGGCAAAC
*entF* ^*d*^	TTCCAGAAACCACGCTGAG	CCCGATAGCTGAACTGGTAAC
*gapA* ^*d*^	CGACCTGTTAGACGCTGATTAC	CGATCAGATGACCGTCTTTCAC

a Primers are listed in the orientation 5′ to 3′. Underlined sequences for mutant construction indicate regions homologous to the gene of interest.

b Used for lambda red mutagenesis.

c Used for Gibson assembly.

d Used for qRT-PCR.

### qRT-PCR.

Strains were grown to mid-log-phase cultures, and RNA was isolated as described above and reverse transcribed into cDNA using iScript (Bio-Rad). qRT-PCR was performed on a QuantStudio 3 PCR machine (Applied Biosystems) using PowerUp SYBR green mastermix (Applied Biosystems). See Text S1 for detailed description.

### Purification of lipocalin-2.

Recombinant human lipocalin-2 (Lcn2), expressed as a glutathione *S*-transferase (GST) fusion protein (a kind gift from Michael Bachman) in XL-1 Gold E. coli protein, was purified in a manner similar to that previously described (59, 60). See Text S1 for detailed description.

### *In vitro* growth competition.

Strains were cultured overnight in M9 medium supplemented with 0.4% glucose at 37°C with aeration and appropriate antibiotic selection. The next day, the OD_600_ was determined for each strain, the strains were OD_600_ matched and then diluted 1:100 into 3 mL of pooled human urine. Where applicable, Lcn2 was added to a final concentration of 25 μg/mL or the vehicle control (25% glycerol) in an equal volume. A final concentration of 0.5% arabinose induced the complemented strains, and ampicillin was added to maintain the plasmid. Input CFU were determined for each strain through drip plating of serial dilutions on plain LB agar and antibiotic selection (chloramphenicol or kanamycin). The strains were then grown overnight with shaking at 37°C, and the output CFU of each strain was determined in the same manner as the input.

The competitive index (CI) was calculated as follows: (mutant output/WT output)/(mutant input/WT input). A log_10_ CI of <0 indicates that the WT outcompetes the mutant, and a log_10_ CI of >0 indicates the mutant outcompetes the WT. When competing the Δ*entB* and Δ*fecA*/Δ*entB* strains, the Δ*entB* strain was “WT” and the Δ*fecA*/Δ*entB* was “mutant.” When competing the Δ*entB* eV (i.e., containing empty vector) and Δ*fecA*/*^+fec^* Δ*entB* strains, the Δ*entB* eV strain was “WT” and the Δ*fecA*/*^+fec^* Δ*entB* strain was “mutant.”

### Murine UTI model.

We used three different mouse strains: CBA/J, C57BL/6 WT, and C57BL/6 *Lcn2*^−/−^ (61). CBA/J mice were purchased from Jackson Laboratories, while C57BL/6 WT and C57BL/6 *Lcn2*^−/−^mice were a kind gift from Michael Bachman and bred in-house. All mice used were female. See Text S1 for detailed description.

### Data availability.

Data are available in NCBI’s Gene Expression Omnibus repository under accession no. GSE188170.
