# Supplementary material for: Ferric Citrate Uptake Is a Virulence Factor in Uropathogenic Escherichia coli
Source: mBio. 2022 May 12;13(3):e01035-22. doi: 10.1128/mbio.01035-22 (PMC9239202; doi:10.1128/mbio.01035-22)
Supplement: TEXT S1 [file mbio.01035-22-s0001.docx]

**Supplemental Methods**

**Bacterial culture conditions and growth curves.**

Clinical UPEC strain HM7 was isolated from an otherwise healthy, young woman with cystitis, and the genome was sequenced in a previous study (1). HM7 was grown in either M9 medium (6 g Na_2_HPO_4_, 3 g KH_2_PO4, 0.5 g NaCl, 1 g NH_2_Cl, 0.1mM CaCl_2_, 1 mM MgSO_4_ per liter) supplemented with 0.4% glucose or Luria-Bertani (LB, 10 g tryptone, 5 g yeast extract, 0.5 g NaCl per liter) broth. Mutants and complemented mutants were maintained with appropriate antibiotics (kanamycin 25 μg/mL, chloramphenicol 25 μg/mL, ampicillin 100 μg/mL, and spectinomycin 50 μg/mL). All growth curves were performed on using a Bioscreen-C Automated Growth Curve Analysis System (Growth Curves USA) over 20 hours, with aeration at 37°C, and OD_600_ readings were taken every hour. For all growth curves, strains were grown in M9 medium overnight with shaking at 37°C, then diluted 1:100 into their indicated medium. Human urine was collected and pooled from at least five healthy female volunteers and filter-sterilized.

**Mutant construction.**

*E. coli* HM7 deletion mutants were made using lambda red mutagenesis (2). Briefly, primers containing regions homologous to the 5’ and 3’ ends of the genes to be deleted amplified either a kanamycin resistance cassette from plasmid pKD4 (2) or a chloramphenicol resistance cassette from plasmid pKD3 (2). Mutants were confirmed via PCR using primers that bind upstream and downstream of the target gene to be deleted and examining for a product size shift compared to WT HM7. All mutants remained marked with antibiotic cassettes. See **Table 3** for all mutant strains, and **Table 4** for all primers used.

**Mutant complementation.**

All complementation vectors were constructed using Gibson assembly (NEB). The *entB* complementation vector was constructed using the promoter-less plasmid pGEN-spec. *entB* and the native *entB* promoter and ribosomal binding site were amplified from WT HM7 gDNA, and then inserted into the backbone of pGEN-spec (position 5333). The pGEN-spec backbone was amplified using PCR.

The *fecABCDE* complementation vector was constructed using pBAD-A, with an arabinose inducible promoter. The *fec* operon was amplified from WT HM7 gDNA and inserted 42 bp downstream of the ribosome binding site within the plasmid (position 352). The plasmid backbone was amplified using PCR.

See **Table 2** for all complemented mutant strains, and **Table 4** for all primers used.

**RNA isolation and library preparation, and sequencing.**

*E. coli* HM7 was cultured overnight in M9 medium, supplemented with 0.4% glucose shaking at 37°C. Overnight cultures were diluted 1:100 in M9 medium with 0.4% glucose supplemented with either 36 μM FeCl_3_ (Sigma**)**, or 150 μM 2,2’ dipyridyl (Sigma**) and grown to mid-log phase (0.4-0.6 OD_600_). Cultures were then treated with Bacterial RNA Protect (Qiagen), harvested by centrifugation and the pellets stored at -80°C. This was performed in biological triplicate.**

**The pellets were treated with lysozyme (Sigma, final concertation: 1 mg/mL) and proteinaseK (Qiagen, final concertation: 1.81 μg/mL) in RNAse-free Tris-EDTA buffer (Corning), for five minutes with continual vortexing. Total RNA was extracted using RNeasy mini kit (Qiagen**)**. Genomic DNA was removed with Turbo DNA-*free* kit (Invitrogen). Bacterial rRNA was removed with the RiboMinus Transcriptome Isolation Kit, Bacteria (Invitrogen). The libraries were prepared using** NEBNext Ultra II Directional RNA Library Prep Kit and sequenced using an Illumina NextSeq-500 (paired end, 38 bp read length).

**qRT-PCR.**

For citrate sensitivity experiments, both WT HM7 and the Δ*entB* strain were grown overnight in M9 medium, supplemented with 0.4% glucose shaking at 37°C. Overnight cultures were back-diluted 1:100 into M9 with 0.4% glucose, and M9 with 0.4% glucose supplemented with 10 μM, 100 μM, 1 mM, 10 mM and 100 mM sodium citrate (Sigma). Cultures were **and grown to mid-log phase (0.4-0.6 OD_600_), treated with Bacterial RNA Protect, pelleted, and stored at -80°C.** Total RNA was isolated and genomic DNA was removed as described above.

RNA (1 μg) was reverse-transcribed into cDNA using iScript cDNA synthesis kit (BioRad). qRT-PCR was performed using 10 ng of template cDNA on a Quantstudio 3 PCR system (Applied Biosystems), with either technical duplicate or triplicate with PowerUp Syber Green mastermix (Applied Biosystems). Samples were analyzed using the 2^-ΔΔCT^ method, with *gapA* as the housekeeping gene. See **Table 3** for primer sequences.

**Purification of Lipocalin-2.**

Recombinant human Lipocalin-2 (Lcn2) expressed as a glutathione S-transferase (GST) fusion protein (3) (a kind gift from Dr. Michael Bachman) in XL-1 Gold *E. coli* protein was purified in a similar manner as previously described (4, 5).

To remove the glutathione from the purified protein the purification was dialyzed overnight at 4°C in 50mM Tris pH 7.5, 100mM NaCl using a 10 kDa cutoff dialysis cassette (Pierce). Simultaneously, the GST tag was cleaved using 50 units of thrombin (Sigma). The next day the protein was run over a glutathione column to remove the cleaved GST-tag, and the flow through, containing the purified Lcn2 was collected. One mL of *p*-Aminobenzamidine–Agarose (Sigma) was added to the Lcn2 to remove the excess thrombin and incubated for one hour at room temperature with head-over-head mixing. The agarose was run over a column, and the flow-through collected. To remove any contaminating pro-Thrombin, the flow through was run over a 50,000 MWCO concentrator columns (Pierce) and the flow-throw was collected. Lcn2 was concentrated at least 5x using 10,000 MWCO concentrator columns (Pierce) and protein concertation was quantified with microplate BCA assay (Pierce). Protein was stored in 25% glycerol at -80°C until use.

**UPEC and fecal genome datasets**

The online bioinformatics resource PATRIC (6) was used to gather UPEC, fecal and environmental *E. coli* strains. UPEC strains were defined as being isolated from patients with UTIs, and specifically were isolated from the urine. Fecal strains were strains isolated from the feces of otherwise healthy patients, and environmental strains were isolated from non-human sites, *e.g*., food sources. **Supplemental Table 1** lists the strain in each of these datasets.

**Murine UTI model**

CBA/J mice were used at 6-8 weeks of age, while the C57B/L6 mice were used at 8 weeks of age. For co-infections mice were inoculated with 50 μL of 2x10^8^ CFU/mL in a 1:1 ratio of WT HM7 and the Δ*fecA* mutant. In mono-infections, mice were inoculated with 50 μL of 2x10^8^ CFU/mL of WT HM7 or mock-infected with an equivalent volume (50 μL) of PBS. Mice were infected using the previously established transurethral model of UTI (7-9)

Input CFU/mL was determined by plating on plain LB and antibiotic (chloramphenicol) agar (Spiral biotech) and colonies counted and CFU enumerated with QCount software. The WT and mutant CFU/mL were determined as described above. The infection progressed for 48 hours, urine was collected and then mice were sacrificed, and bladder and kidneys aseptically removed. The organs were homogenized in sterile PBS then plated (Spiral Biotech) on plain and chloramphenicol-containing LB agar for co-infections and only plain LB for mono-infections. Colonies were counted and bacterial burden was enumerated using Qcount Software; log_10_ CI calculated as described above to assess the relative fitness of each strain.

**Lipocalin-2 ELISA**

Murine LCN was quantified via ELISA (Biolegend). Mice were infected in a mono-infection with WT HM7 as described above. One mL of organ homogenate from either the bladder or the kidney was centrifuged at max speed (13,300 RPM) on a benchtop centrifuge at 4°C for ten minutes to pellet tissue. The supernatant was collected, diluted 1:2 (bladders) or 1:10 (kidneys), or undiluted for mock-infected samples. Lcn2 was quantified using manufacturer’s instructions, and the values normalized to organ mass.

1. Sintsova A, Frick-Cheng AE, Smith S, Pirani A, Subashchandrabose S, Snitkin ES, Mobley H. 2019. Genetically diverse uropathogenic *Escherichia coli* adopt a common transcriptional program in patients with UTIs. eLife 8:e49748.

2. Datsenko KA, Wanner BL. 2000. One-step inactivation of chromosomal genes in *Escherichia coli* K-12 using PCR products. Proceedings of the National Academy of Sciences 97:6640.

3. Bachman Michael A, Oyler Jennifer E, Burns Samuel H, Caza M, Lépine F, Dozois Charles M, Weiser Jeffrey N, Bäumler AJ. 2011. Klebsiella pneumoniae Yersiniabactin Promotes Respiratory Tract Infection through Evasion of Lipocalin 2. Infection and Immunity 79:3309-3316.

4. Bachman MA, Miller VL, Weiser JN. 2009. Mucosal Lipocalin 2 Has Pro-Inflammatory and Iron-Sequestering Effects in Response to Bacterial Enterobactin. PLOS Pathogens 5:e1000622.

5. Bundgaard JR, Sengelov H, Borregaard N, Kjeldsen L. 1994. Molecular Cloning and Expression of a cDNA Encoding NGAL: A Lipocalin Expressed in Human Neutrophils. Biochemical and Biophysical Research Communications 202:1468-1475.

6. Davis JJ, Wattam AR, Aziz RK, Brettin T, Butler R, Butler RM, Chlenski P, Conrad N, Dickerman A, Dietrich EM, Gabbard JL, Gerdes S, Guard A, Kenyon RW, Machi D, Mao C, Murphy-Olson D, Nguyen M, Nordberg EK, Olsen GJ, Olson RD, Overbeek JC, Overbeek R, Parrello B, Pusch GD, Shukla M, Thomas C, VanOeffelen M, Vonstein V, Warren AS, Xia F, Xie D, Yoo H, Stevens R. 2020. The PATRIC Bioinformatics Resource Center: expanding data and analysis capabilities. Nucleic Acids Research 48:D606-D612.

7. Hagberg L, Engberg I, Freter R, Lam J, Olling S, Svanborg Eden C. 1983. Ascending, unobstructed urinary tract infection in mice caused by pyelonephritogenic Escherichia coli of human origin. Infect Immun 40:273-83.

8. Hagberg L, Hull R, Hull S, Falkow S, Freter R, Edén CS. 1983. Contribution of Adhesion to Bacterial Persistence in the Mouse Urinary Tract. Infection and Immunity 40:265-272.

9. Frick-Cheng AE, Sintsova A, Smith SN, Krauthammer M, Eaton KA, Mobley HLT. 2020. The Gene Expression Profile of Uropathogenic *Escherichia coli* in Women with Uncomplicated Urinary Tract Infections Is Recapitulated in the Mouse Model. mBio 11:e01412-20.
